# Age Gelation in Direct Steam Infusion Ultra-High-Temperature Milk: Different Heat Treatments Produce Different Gels

**DOI:** 10.3390/foods13081236

**Published:** 2024-04-18

**Authors:** Peipei Wu, Mengyuan Guo, Pengjie Wang, Yi Wang, Ke Fan, Hui Zhou, Wentao Qian, Hongliang Li, Menghui Wang, Xiaojun Wei, Fazheng Ren, Jie Luo

**Affiliations:** 1 College of Food Science and Technology, Hunan Agricultural University, Changsha 410114, China; wu15638612862@163.com (P.W.); 15270023196@163.com (K.F.); zhouhui@hunau.edu.cn (H.Z.); 2 Key Laboratory of Functional Dairy, Co-Constructed by Ministry of Education and Beijing Government, College of Food Science and Nutritional Engineering, China Agricultural University, Beijing 100083, China; guomengyuan@cau.edu.cn (M.G.); wpj1019@cau.edu.cn (P.W.); 3 College of Food Science and Engineering, Tianjin University of Science & Technology, Tianjin 300457, China; wangyi922217@126.com; 4 Mengniu Hi-Tech Dairy Products (Beijing) Co., Ltd., Beijing 101100, China; qianwentao2006@163.com (W.Q.); lihongliang@mengniu.cn (H.L.); 5 Inner Mongolia Mengniu Dairy (Group) Co., Ltd., Hohhot 011500, China; wangmenghui@mengniu.cn (M.W.); weixiaojun@mengniu.cn (X.W.)

**Keywords:** direct UHT, age gelation, gel property, gel structure, interaction force

## Abstract

To investigate the gelation process of direct ultra-high-temperature (UHT) milk, a pilot-scale steam infusion heat treatment was used to process milk samples over a wide temperature of 142–157 °C for 0.116–6 s, followed by storage at 4 °C, 25 °C, and 37 °C. The results of the physicochemical properties of milk showed that the particle sizes and plasmin activities of all milk samples increased during storage at 25 °C, but age gelation only occurred in three treated samples, 147 °C/6 s, 142 °C/6 s, and 142 °C/3 s, which all had lower plasmin activities. Furthermore, the properties of formed gels were further compared and analyzed by the measures of structure and intermolecular interaction. The results showed that the gel formed in the 147 °C/6 s-treated milk with a higher C* value had a denser network structure and higher gel strength, while the 142 °C/6 s-treated milk had the highest porosity. Furthermore, disulfide bonds were the largest contributor to the gel structure, and there were significant differences in disulfide bonds, hydrophobic interaction forces, hydrogen bonds, and electrostatic force among the gels. Our results showed that the occurrence of gel was not related to the thermal load, and the different direct UHT treatments produced different age gels in the milk.

## 1. Introduction

In direct ultra-high-temperature (UHT) sterilization, milk is directly mixed with steam under pressure and then subjected to vacuum cooling. This heating treatment greatly decreases the thermal load compared with indirect UHT treatment [1]. Therefore, more bioactive components in the raw milk are retained [2]. However, direct UHT milk often suffers from instability with age gelation, sedimentation, and creaming during storage [3]. In age gelation, liquid milk forms a three-dimensional protein network during storage that increases the viscosity and causes the milk to lose fluidity. The formed gel is usually weak and initially tends to form at the bottom and extend throughout the pack over time. This phenomenon in direct UHT milk is mainly attributed to plasmin [4,5].

Plasmin, an endogenous enzyme, is a component of a complex system consisting of plasmin, plasminogen, plasminogen activator, plasminogen activator inhibitor, and plasminogen inhibitor [6,7]. Among them, two inhibitors are sensitive to direct UHT treatment and even almost completely inactivated, while plasmin, plasminogen, and plasminogen activator are thermostable. They can be retained to a greater extent [8]. Thus, plasminogen is continuously converted to plasmin by plasminogen activator during storage. Consequently, plasmin will hydrolyze β-casein (β-CN) and α_S1_-casein (α_S1_-CN)/α_S2_-casein (α_S2_-CN) inside casein micelles, causing the destabilization of casein micelles and the release of tendrils composed of κ-casein (κ-CN) and β-lactoglobulin (β-Lg), and then these tendrils anchor with each other to ultimately form a gel [3,5]. On the other hand, if residual plasmin-induced proteolysis occurs too rapidly, this protein system does not have time to rearrange to form a gel; thus, clarification/sedimentation of the milk may occur rather than age gelation [3].

For the age gelation by plasmin in UHT milk, indirect UHT milk was once the main subject of research, but with the application of direct UHT technology in liquid milk processing, protein destabilization during storage still exists. Heating units for direct UHT treatment consist of two types: either an injection type, in which steam is injected into the product, or an infusion type, in which the product is infused into a chamber of steam [9]. The infusion method is gentler than that of steam injection and does not cause disruption of fat globules [9]. Malmgren et al. [5] found that the steam injection method produced more sediment than steam infusion under the same UHT treatment (140 °C/4 s) and storage conditions. Thus, it is of vital importance to study the mechanisms of age gelation in direct steam infusion UHT milk. However, many heating conditions in previous studies were limited in range, such as 140–150 °C for 3–6 s [5,10,11,12,13,14]. Given that direct steam infusion UHT treatment can achieve extremely short heating time (<0.12 s), we designed a wide temperature range of 142–157 °C for 0.116–6 s in this study. It should be noted that the extreme condition of the equipment used in this study is 0.116 s for heating, and commercial direct UHT milk in China has a similar heating time. In addition, previous studies have mostly focused on the protein behavior of bovine milk before age gelation, but there has been limited research into the appearance and development of aged gel. In our previous study, we investigated the composition of aged gel in direct steam infusion UHT milk by using Sodium dodecyl sulfate–polyacrylamide gel electrophoresis (SDS-PAGE) and peptidomics analysis, where we found that the aged gel is primarily composed of intact β-Lg, β-CN, α_S2_-CN, α_S1_-CN, κ-CN, α-lactalbumin, and peptides derived from α_S2_-CN, α_S1_-CN, and β-CN. Additionally, the presence of other proteins and peptides in milk may contribute to gel formation. However, it is not clear which action dominates the gel formation or whether the gel structure is consistent across different direct UHT conditions.

Therefore, this study aimed to investigate the gelation process of direct UHT milk treated with steam infusion over a wide temperature range of 142–157 °C for 0.116–6 s and then stored at 4 °C, 25 °C, or 37 °C, respectively. The changes in the pH, particle size, zeta-potential, and plasmin activity of the milk after direct UHT treatment and during storage were monitored. To further investigate the characteristics of gels produced in the milk samples, the gel strength, macrostructure, and microstructure, and the contribution of intermolecular interaction forces of gels were compared and analyzed. The study provides guidance for the optimization of heat treatment and storage parameters for direct steam infusion UHT milk.

## 2. Materials and Methods

### 2.1. Milk Samples

Skim milk was obtained from the local industry in Maanshan, China. Bacteria in the raw milk were at low levels of 0.6–2.2 × 10^4^ CFU/mL and somatic cell counts of 12.3–35.1 × 10^4^ CFU/mL. The milk was transported to SPX (Shanghai) FLOW Technology Co., Ltd. (Shanghai, China) via a 4 °C cold chain for follow-up pilot-scale commercial direct UHT treatment. A total of 500 L of skim milk was pasteurized at 72 °C for 25 s. Subsequently, the skim milk was heated by a direct UHT system (InfusionPlus^TM^, SPX Flow, Charlotte, NC, USA) with a steam infusion chamber (direct UHT conditions: 142 °C for 0.25 s, 3 s, or 6 s; 147 °C for 0.25 s, 3 s, or 6 s; 153 °C for 0.116 s, 0.25 s, or 6 s; or 157 °C for 0.116 s; 50 L of skim milk was processed for each of the heat treatments). Thereafter, the product was cooled by vacuum cooling back to 75 °C and then directed to the two-stage homogenizer (15 MPa). Finally, the product was cooled indirectly to 20 °C and collected in an aseptic storage tank. The direct UHT-treated skim milk for each heat treatment was filled aseptically into 50 plastic containers (350 mL; 6 cm diameter). Samples were stored at 4 °C, 25 °C, or 37 °C and analyzed at intervals of approximately half a month. Once sedimentation or age gelation occurred, the storage was discontinued. Before each analysis, the sample is fully mixed by inversion. Immediately before storage, the changes in pH value, particle size, and zeta-potential of the samples before and after direct UHT treatment were analyzed. Information regarding heating conditions and storage temperatures is shown in Supplementary Materials (Table S1). C* provides a measure of the effect of a heat treatment on the chemical components of a product. In addition, C* can be calculated by the following equation:

C*=∫10T−13531.4∗dt30.5

where T is the heating temperature, and t is time [9].

### 2.2. Physicochemical Properties of Milk during Storage

#### 2.2.1. Total Plate Count

The total bacterial count of milk samples was determined by using the test piece (Food Safety Tech., Food Safety Technology Co., Ltd., Guangzhou, China) according to the manufacturer’s instructions. This test piece contains a color-developing reagent, a soluble absorbent gel, and the same nutrients as Plate Count Agar medium. Briefly, 1 mL of the sample was fully mixed with 9 mL of sterile normal saline to obtain the 1:10 solution of the sample. Then, 1 mL of the 1:10 solution was fully mixed with 9 mL of sterile normal saline to obtain the 1:100 solution of the sample. We dropped 1 mL of the solution into the test piece and operated with two test pieces for each dilution. Finally, the test pieces were incubated at 37 °C for 24 h [15].

#### 2.2.2. pH Value

Skim milk samples stored at 4 °C, 25 °C, and 37 °C were either heated by a water bath (Bluepard; Shanghai, China) or cooled by an ice water bath (HMKXYQ; Wuxi, China) to ensure temperature balance. The pH value of the milk was measured at room temperature (25 °C) using a pH meter (Sevencompact, Shanghai, China).

#### 2.2.3. Particle Size and Zeta-Potential

According to the methodology described by Li et al. [16], the Malvern Zetasizer Nano ZEN3600 instrument (Malvern Instruments Ltd., Malvern, UK) was used to determine the size and zeta-potential of milk proteins. To determine the particle size, the samples were diluted with ultra-pure water (1:200) and subsequently transferred into a cuvette (DTS0012, Malvern Instruments Ltd., Malvern, Worcestershire, UK). All measurements were performed at 25 °C using the refractive index of the milk protein: 1.45. The measurements were performed at a fixed angle of 173°, with each measurement consisting of 11 runs for 15 s. 

#### 2.2.4. Plasmin Activity

According to the methodology described by Rauh et al. [11] with some modifications, plasmin-derived activity was measured by the rate of hydrolysis of the chromogenic substrate S-2251 (Chromogenix, Roncello, Italy) for plasmin. Briefly, 1 mL of milk sample was mixed with 250 μL of trisodium citrate buffer (0.4 M; pH = 8.9; Merck, Shanghai, China), followed by a vortex for 15 min to dissociate casein micelles. Then, an equal volume of the detection buffer (0.1 M Tris-HCl, 8 mM 6-Aminocaproic acid, 0.4 M NaCl; pH = 8) was added to the milk sample–buffer mixture. After that, 75 μL of Substrate S-2251 solution was mixed with 75 μL of the mixture and incubated at 37 °C and detected at 20 min intervals over 120 min. The absorbances were measured using a microplate reader. A milli-unit (mU) refers to the amount of plasmin that catalyzes the conversion of 1 nanomole of S-2251 per minute.

### 2.3. Gel Analysis

#### 2.3.1. Determination of Gelation Time Range

To determine the occurrence of age gelation in the sample during storage, the milk samples during each storage period were sifted with a 100-mesh sieve complying with U.S. standards, corresponding to sieve opening dimensions of 150 µm [17]. When there were particles that could not pass through the sieve, we determined that the milk produced aged gel at this time range.

#### 2.3.2. Dynamic Rheological of Gel

The supernatant was carefully aspirated from the plastic container; then, the plastic container was cut to collect the intact gel. Dynamic rheological properties of the gel’s samples were tested using an AR1500 (TA Instruments, New Castle, DE, USA). A stainless-steel parallel plate (40 mm in diameter) was used, and the gap was set at 1 mm. Strain sweeps were performed over the range of 0.01% to 10% strain at a frequency of 1 Hz to determine the stress value. After that, a frequency scan on the other gel sample was performed. The angular frequency was changed from 0.628 to 6.283 rad/s at 1% strain, which was within the linear viscoelasticity region. The data points were recorded at a rate of 10 points per decade [18]. The G’ (storage modulus) and variation trend of the loss angle (tan δ = G’’/G’) with frequency were also obtained in the experiment.

#### 2.3.3. Water Holding Capacity

Water holding capacity was calculated by dividing the weight of gel after centrifugation (1000× *g* for 10 min; 4 °C) by the weight of gel (1 g) before centrifugation [19].

#### 2.3.4. Observation of Gel Microstructure

A confocal laser scanning microscopy (A1Rsi, Nikon Instruments, Tokyo, Japan) with a ×63 oil immersion objective was applied to observe the distribution of protein in the formed gel. Protein phases of gels were stained with Fast Green solution (Sigma-Aldrich, Saint Louis, MO, USA. 0.1 mg/mL in water). Overall, 1 mL of the sample was mixed with 50 μL Fast green. The stained sample was kept in the dark for 10 min at room temperature (25 °C) to ensure complete labeling. The excitation wavelength of the Fast Green was 633 nm [20].

#### 2.3.5. Gel Porosity

The porosity of milk gel was analyzed by captured confocal laser scanning microscopy images. The captured image is converted into an 8-bit image for analysis. Image J software (1.8.0, National Institutes of Health, Bethesda, MD, USA) is used to perform grayscale image analysis. The porosity of the gel is expressed as an area fraction percentage, which is defined as the ratio of the pixels contributed by the pores in the image to the total number of pixels in the image [21].

#### 2.3.6. Intermolecular Interactions

The solubility of the gel was analyzed according to the method described by Zhao et al. and Ran et al. [22,23] with some modifications. The sodium chloride solution (0.6 M; Merck, Shanghai, China) could disrupt ionic bonds, 1.5 M urea (Merck, Shanghai, China) could destroy hydrogen bondings, 8 M urea could simultaneously destroy the hydrogen bondings and hydrophobic interactions, and 0.5 M 2-Hydroxy-1-ethanethiol (Merck, Shanghai, China) could disrupt disulfide bonds. Gels were successively solubilized in four solvents: 0.6 M sodium chloride (S1); 0.6 M sodium chloride + 1.5 M urea (S2); 0.6 M sodium chloride + 8 M urea (S3); and 0.6 M sodium chloride + 8 M urea +0.5 M 2-Hydroxy-1-ethanethiol (S4). Approximately 3 g of gel were mixed with 27 mL of S1, and then the mixture was dispersed using a high-speed disperser (FJ200-SH, Shanghai Specimen Model Ltd., Shanghai, China) at 10,000 rpm for 1 min, followed by centrifugation at 10,000× g for 25 min. The precipitate obtained from S1 was homogenized in 27 mL of S2 and then subjected to the same procedure. The same procedure was also successively performed using S3 and S4. The protein concentration was determined by the Bradford Protein Assay Kit (Beijing Solarbio Science & Technology Co., Ltd., Beijing, China) according to the manufacturer’s instructions. The results presented are the average of three measurements and are expressed as the percentage of each fraction concerning total protein.

### 2.4. Statistical Analysis

All measurements were performed in biological triplicates, and the results are expressed as means and standard deviations. We analyzed data using one-way repeated measures ANOVA in SPSS (SPSS 19.0, SPSS Inc., Chicago, CA, USA). Significant differences (*p* < 0.05) were analyzed using Duncan’s multiple-range test. GraphPad Prism software (Version 8.01; San Diego, CA, USA) was used to visualize the data.

## 3. Results and Discussion

### 3.1. Effects of Direct UHT Treatment on the Physicochemical Properties of Milk

Changes in the pH value, particle size, and zeta-potential of milk treated with different direct UHT treatment conditions are shown in Table 1. After direct UHT treatment, the pH value of the milk decreased. This trend is mainly due to an insolubilization of calcium phosphate, which migrates to the colloidal phase, consequently decreasing the concentration of phosphate ions in the whey [24]. Upon direct UHT treatment, the particle size of the milk increased significantly (*p* < 0.05). The attachment of denatured whey proteins to casein micelles caused by heating may contribute to increasing milk particle size [11]. The homogenization pressure of 15 MPa set based on the actual operating parameters of the UHT equipment may not be high enough to disrupt the heating-induced association between casein micelles, which may also increase the size of milk [11,25]. Moreover, C* in this study provides a measure of the effect of direct UHT treatment on the chemical components of the milk. We found that samples with a high C* value (>0.1) had smaller particle sizes, suggesting heat-induced dissociation of κ-CN from the casein micelle during direct UHT treatment [12]. The zeta-potential value did not change much and only slightly due to the association of β-Lg with κ-CN at the surface of casein micelles.

### 3.2. Non-Gelation Observation of Direct UHT Milk Stored at Different Temperatures

The gelation process was monitored for direct UHT skim milk treated in different conditions and stored at 4 °C, 25 °C, and 37 °C. The storage stability of milk was best at 4 °C, and no age gelation or obvious sedimentation occurred even after 6 months of storage at 4 °C. By contrast, the severe protein destabilization of milk stored at 37 °C was observed. The milk samples treated at 153 °C, 147 °C, and 142 °C for 0.25 s and 157 °C and 153 °C for 0.116 s showed obvious sedimentation and no gel formation within 1 month of storage at 37 °C. All other groups of samples produced sediments during the later period of storage, and images of all samples are shown in Supplementary Materials (Figure S1). In addition, no formed gel was observed at 37 °C. The appearance of sedimentation may be because the optimum temperature of plasmin is approximately 37 °C; consequently, plasmin-induced proteolysis occurred too rapidly, and the protein system did not have time to rearrange to form a gel [26]. Therefore, samples with sedimentation will not be determined in subsequent experiments.

### 3.3. Characteristics of Direct UHT Milk Stored at 25 °C

#### 3.3.1. Gel Appearance

**Figure 1:**
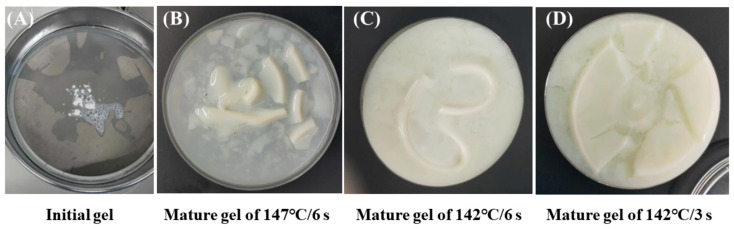
The apparent structure of the initial gel of 142 °C/3 s (**A**); and the age-gel formed in milk with direct UHT treatments after storage at 25 °C (**B**–**D**). (**B**) Mature gel of 147 °C/6 s samples stored for 4 months; (**C**) Mature gel of 142 °C/6 s samples stored for 5 months; (**D**) Mature gel of 142 °C/3 s samples stored for 3.5 months.

To monitor the appearance and development of gel at 25 °C, the milk samples during storage were filtered regularly with a 100-mesh sieve. This study found that residue on a 100-mesh screen can pass through the screen due to the friction and breaking of the particles. These substances were the initial gels which were soft and fragile (Figure 1A). Within half a month after the occurrence of the phenomenon in Figure 1A, the milk began to form a visible gel-like substance at the bottom of the bottle, and flocculent gel fragments were observed when gently shaking the bottle. The gels formed by samples from the 147 °C/6 s, 142 °C/6 s, and 142 °C/3 s treatment groups stored at 25 °C for 4 months, 5 months, and 3.5 months, respectively, are shown in Figure 1B–D. The results showed that the morphology of the gel was dependent on heat-induced chemical changes. Samples treated with two direct UHT conditions (147 °C/6 s and 142 °C/6 s) have higher C* values, and relatively firm gels were formed. In contrast, 142 °C/3 s treatment had a lower C* value, and relatively loose gels were formed. The difference in the morphology of the formed gel was due to the fact that a higher thermal load induces more and longer tendrils, which contributed to forming a relatively firm state [5]. To further analyze why only three groups of samples underwent aging gelation while the other treatment groups underwent sedimentation, we monitored the changes in pH value, particle size, zeta-potential, and plasmin activity during storage.

#### 3.3.2. Physicochemical Properties of Direct UHT Milk Stored at 25 °C

##### pH Value

**Figure 2:**
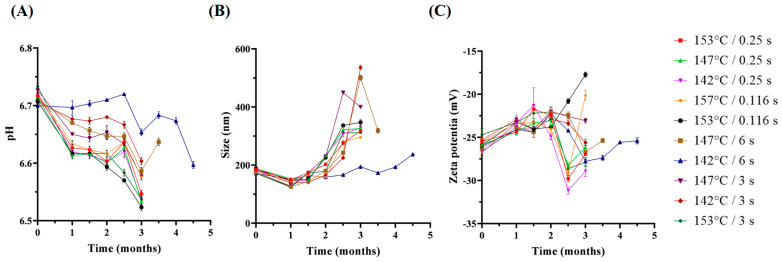
Changes in (**A**) pH value, (**B**) particle size, and (**C**) zeta-potential of direct UHT milk samples during storage at 25 °C.

The overall trends for the pH values in all samples were relatively similar (Figure 2A). Generally, the pH value of the milk decreased during storage, which was consistent with previous studies [27]. The decrease in pH value is likely caused by the formation of acids from lactose and the dephosphorylation of the amino acids PSer and PThr present in caseins [28,29]. The fluctuation in the pH value during storage may be caused by the dephosphorylation of casein or proton release caused by casein hydrolysis by plasmin [30].

##### Particle Size and Zeta-Potential

The particle sizes in the milk during storage at 25 °C are shown in Figure 2B. During the first month of storage at 25 °C, the particle size of the milk proteins decreased. It suggested that the rate of hydrolysis of casein by plasmin to produce small-sized proteins in the early stages of storage might be greater than the rate at which the hydrolyzed casein fragments bind to each other. During the middle stage of storage, the particle sizes of the milk samples started to increase, probably because more calcium and calcium phosphate bridges formed between caseins and casein sub-micelles, inducing the larger casein micelles [16]. A sharp increase in protein particle sizes for the 147 °C/6 s and 142 °C/3 s samples stored at 25 °C for 3 months may be caused by the association between peptides produced by plasmin-induced hydrolysis and micelle fragments via hydrophobic bonding and calcium bridge interactions to form a three-dimensional gel network [3,8].

The zeta-potential changes during storage are shown in Figure 2C. Over the longer storage period, the absolute zeta-potential first decreased and then increased. A reduction in the absolute value of the zeta-potential during storage probably occurred because of the association between casein micelles [16,31]. However, the increase in absolute zeta-potential values of the milk samples during storage may be due to the release of the β-Lg-κ-CN complex from casein micelles induced by plasmin. Thus, it is assumed that the increased electrostatic interactions affect gel formation rather than sedimentation.

#### 3.3.3. Plasmin Activity in Direct UHT Milk Stored at 25 °C

Although microbial enzymes (produced mainly by psychrotrophic bacteria) are very heat-resistant, which may remain active under direct UHT treatment, the gel formed in this study was most likely not due to microbial action. There were few microorganisms in the raw milk (total bacterial count: 0.6–2.2 × 10^4^ CFU/mL), and the heat treatment would reduce the number of enzymes produced by microorganisms. The raw milk used in this experiment was pasteurized at 75 °C/25 s and subjected to high heat treatment at >142 °C to kill the microorganisms. A total number of colonies results indicated that no microorganisms were detected in all samples during storage (results not shown). These results also indicated that there was no production of microbial enzymes during storage. Therefore, the contribution of bacterial proteases should be low. Meanwhile, the gel formed in the three samples was soft and fragile (Figure 1). Zhang et al. [20] compared the gel produced by exogenous enzymes and plasmin for hydrolyzing milk protein. Their results showed that the gel structure from plasmin was loose and fragile, while the gel from exogenous enzymes was harder and less fragile. Therefore, in this study, we focused on changes in the plasmin activity of the milk samples during storage.

**Figure 3:**
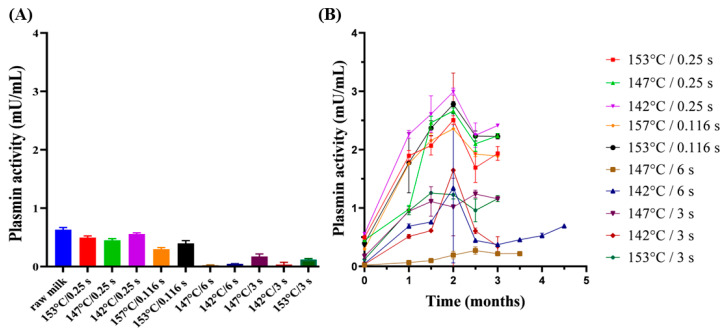
Changes in plasmin activity in (**A**) milk before and after direct UHT treatment; (**B**) direct UHT milk samples during storage at 25 °C.

The milk samples with a low C* value (<0.1) have higher initial plasmin activity and vice versa. During storage at 25 °C, the plasmin activity increased (Figure 3B) for all sample groups. The five groups with high temperature/short holding time treatment had higher plasmin activity and a larger increase in activity than the high temperature/long holding time treatment group. This could be because plasminogen and plasminogen activators have strong heat resistance and can survive under direct UHT treatment. The residual plasminogen will be converted into plasmin, consequently increasing the plasmin activity [32]. After two months, the plasmin activities of samples started to decrease. This was possible due to the activation and/or inhibition of components of the complex plasmin system and even the degradation of plasmin [14]. The plasmin activity of the aged gel group samples remained at a low level during storage (Figure 3B). This result indicated that limited residual plasmin activity leading to limited hydrolysis of casein may be the cause of gel formation. When plasmin activity is very high, it tends to cause sedimentation; therefore, a higher plasmin activity may not make it easier to form a gel. Instead, a moderate level of hydrolysis will lead to gel formation, which is consistent with what is mentioned in the literature.

The differences in plasmin activity and whey protein denaturation caused by different heat treatment conditions may be the reasons for the differences in the macrostructure of the three groups of gels. Studies have shown that a reasonable increase in protein concentration will increase the strength of protein gel [33]. The complex formed by cross-linking of denatured whey protein and casein accumulates at the bottom of the milk bottle because of sedimentation, increasing the protein concentration at the bottom compared with other layers [34,35,36]. However, an appropriate hydrolysis intensity will allow sufficient time for the hydrolyzed casein to form a complex and sediment at the bottom of the bottle. This will result in a high concentration of milk protein at the bottom of the bottle and facilitate gel formation.

### 3.4. Gel Characterization

#### 3.4.1. Gel Microstructure and Porosity Ratio

**Figure 4:**
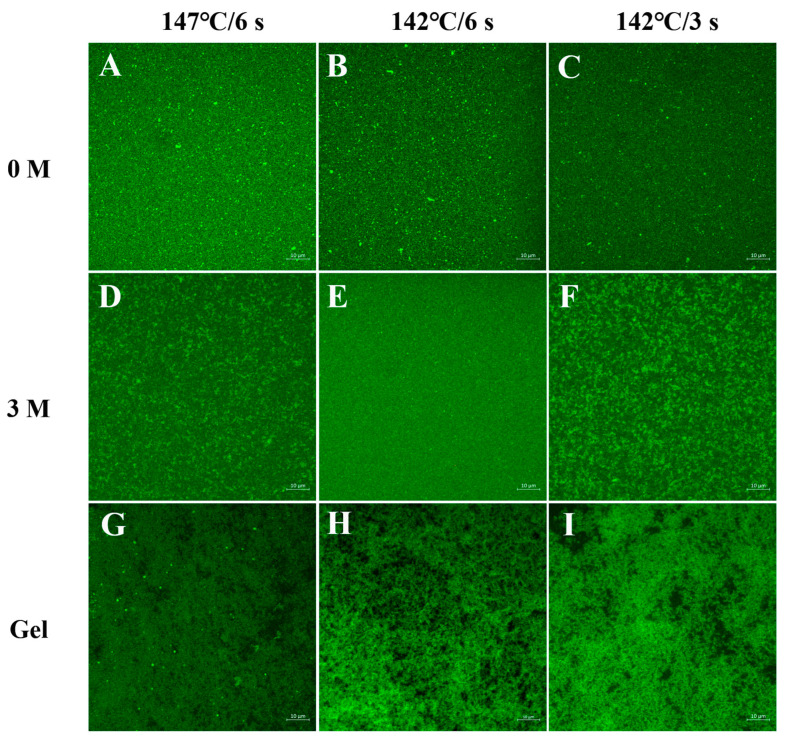
Microstructure of direct UHT milk samples during storage at 25 °C. Microstructure of milk at the beginning of storage (**A**–**C**), after 3 months (**D**–**F**), and microstructure of age-gels formed when they reached the gel time range (**G**–**I**). (**A**,**D**,**G**), (**B**,**E**,**H**) and (**C**,**F**,**I**) refer to milk treated at 147 °C/6 s, 142 °C/6 s, and 142 °C/3 s, respectively. The protein is dyed green. The scale bars are 10 μm in length.

Changes in the microstructures of milk samples during storage at 25 °C were observed (Figure 4). Protein particles are evenly distributed in milk after direct UHT treatment (Figure 4A–C). In the middle and later stages of storage, the confocal images of the 147 °C/6 s and 142 °C/3 s samples showed obvious protein aggregation and slight cross-linking (Figure 4D–F). These changes could be the reason for the sudden increase in particle size. The aggregated proteins in the gel were cross-linked to form a gel network (Figure 4G–I). The gel network formed by the 147 °C/6 s sample had small pores and a dense structure, and large protein aggregates directly participated in the formation of the gel network. The gel network formed at 142 °C/6 s was loose and porous and had the largest pores. The pore size of the gel network formed at 142 °C/3 s was between those of the 147 °C/6 s and 142 °C/6 s samples.

The porosities of the three groups of gel samples are shown in Table 2. The gel network structure formed in the 147 °C/6 s sample was dense, and the porosity of the gel was 14.30% ± 0.76%. The gel network formed in the 142 °C/6s sample had many large pores, and the highest porosity was 42.43% ± 0.99%. The gel network structure formed in the 142 °C/3 s sample was denser than that of the 142 °C/6 s sample, and the porosity was 21.49% ± 2.25%. The differences in whey protein denaturation and plasmin activity caused by different direct UHT treatment conditions may be the main reasons for the differences in microstructures of the gels.

#### 3.4.2. Dynamic Rheological Analysis

**Figure 5:**
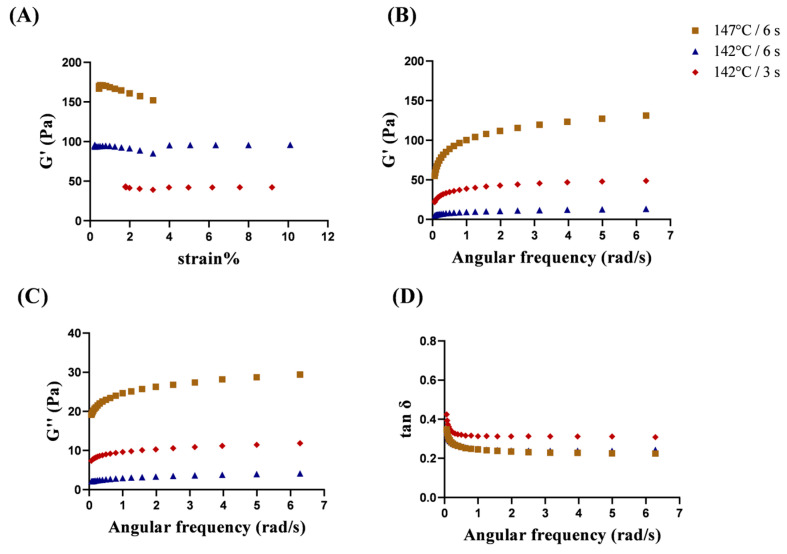
Rheological properties of age-gels formed in different direct UHT milk samples after storage at 25 °C. (**A**) steady-state shear stress scans; (**B**) elastic modulus (G′); (**C**) viscous modulus (G″); and (**D**) tan δ.

None of the gels deformed significantly at 1% stress (Figure 5A). Therefore, modulus analysis was carried out at 1% stress. Changes in the elastic modulus were measured for the three sets of gel samples (Figure 5B). The most elastic gel formed with 147 °C/6 s treatment, and the least elastic gel formed with 142 °C/6 s treatment. This is related to the network structure of the gel formed by coagulation. The gel formed with 147 °C/6 s treatment had smaller pores and finer clusters than the gels from the other two treatments, which meant that the structure was less likely to be deformed under the same stress and showed better elasticity and gel strength [18]. The microstructures of the three groups of gel samples (Figure 5) also support the results for the elastic modulus. The values of tan *δ* of all samples decreased as the frequency increased (Figure 5D). The tan *δ* of all gel samples were all <1, which indicated that the gel exhibited solid elasticity [37]. The results confirmed that, the gels formed under different direct UHT treatment conditions were very different. Therefore, control of thermal parameters will greatly affect the storage quality of the product.

#### 3.4.3. Water Holding Capacity Analysis

The water holding capacity of the gels produced by the different direct UHT milk is shown in Table 2. The gel formed with 147 °C/6 s treatment had the greatest water retention. This may be related to the network structure of the gel. By comparison, the gel formed with 147 °C/6 s treatment had a denser network structure (Figure 4G). The retained water in this gel network would not be readily lost with the destruction of the network structure during centrifugation. Consequently, the gel water-holding capacity was as high as 95.1 ± 0.4%. For the 142 °C/6 s sample, the gel structure was loose, the interaction between protein molecules was weak, and the porosity was high. After centrifugation, the network structure was readily destroyed, and the water retention capacity was poor (water holding capacity: 59.9 ± 1.8%). The gel strength of the 142 °C/3 s sample was between that of the other two samples, and the porosity was 79.5 ± 3.4% [18]. The water-holding capacity results are consistent with the gel network porosity results.

#### 3.4.4. Intermolecular Interaction Analysis

**Figure 6:**
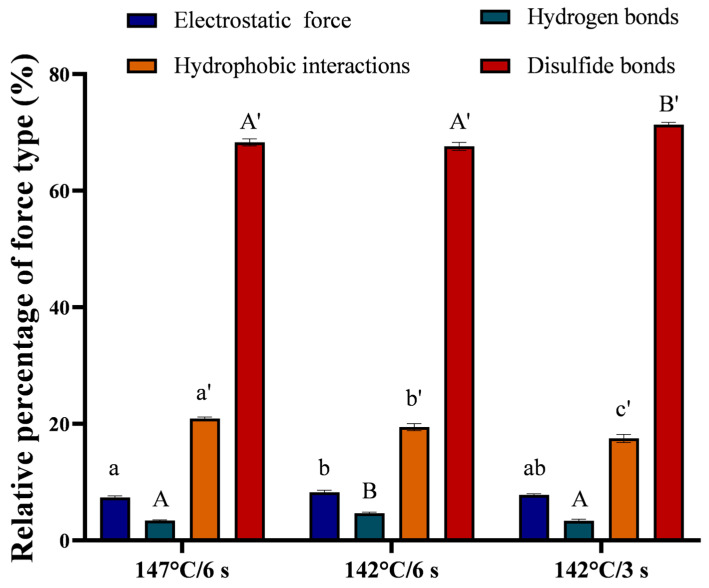
Contribution of intermolecular interaction forces in the network structure of age-gels in direct UHT milk with different heat treatments after storage at 25 °C. Each value represents the mean ± standard deviation (*n* = 3). Values with different letters in the same column differ significantly (*p* < 0.05).

The occurrence of a gel network structure in direct UHT milk is a result of protein aggregation after plasmin hydrolysis, which leads to covalent (disulfide bond) and non-covalent interactions (ionic bonds, hydrogen bonds, and hydrophobic interactions) [3,8]. To further explore the key force maintaining the three-dimensional network structure of the gel in milk, the type and percentage of intermolecular forces were determined. As shown in Figure 6, the key force was disulfide bonds, followed by hydrophobic interactions. This result was similar to those of Duan et al. [38] who reported that disulfide bonds and hydrophobic interactions were positively correlated with the gel strength. In addition, the sum of the hydrophobic interaction force and disulfide bond contribution of the sample at 147 °C/6 s was larger than that of the 142 °C/6 s sample (Figure 6), which was consistent with the results in Figure 5B. Additionally, there were significant differences among the contributions of hydrophobic interactions to the maintenance of the gel network structure among the three groups of gel samples. The contribution of hydrophobic interactions in the 147 °C/6 s sample was greater than that in the 142 °C/6 s or 142 °C/3 s sample. These differences may be related to the degree of protein denaturation and unfolding caused by heat treatment [39]. There was a significant difference in the contributions of electrostatic interactions between the 147 °C/6 s and 142 °C/6 s samples (*p* < 0.05). This was consistent with the results in Figure 2C. Furthermore, there was a significant difference in the contributions of hydrogen bonding between the 147 °C/6 s and 142 °C/3 s samples and between the 142 °C/3 s and 142 °C/6 s samples (*p* < 0.05). However, the contributions of electrostatic interactions and hydrogen bonding were smaller and mainly dominated by disulfide bonds and hydrophobic interactions. Combined with the plasmin activity results, we speculate that the gel that appears in direct UHT milk during storage is mainly caused by plasmin hydrolyzing casein and releasing sulfhydryl groups buried in the protein molecules. When the activity of residual plasmin is high, direct UHT milk tends to form a protein sediment rather than a gel because of excessive casein hydrolysis. 

## 4. Conclusions

**Figure 7:**
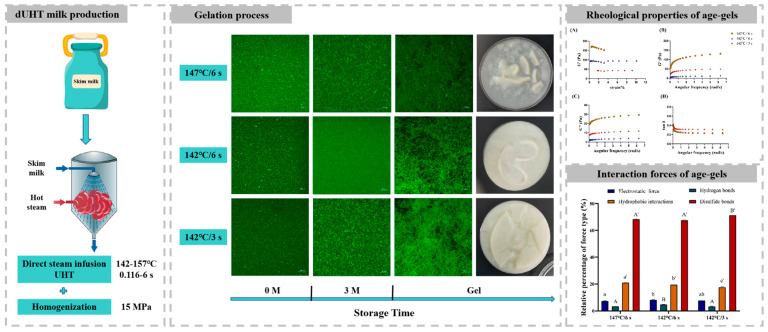
Simplified scheme describing formation mechanisms of aging gel in direct UHT skim milk.

The effect of direct steam infusion UHT treatment on milk was monitored for the gelation process of the milk during storage. Milk stored at 4 °C remained stable for 6 months, while milk stored at 37 °C rapidly produced a protein sediment. Although the particle sizes and plasmin activities of the milk samples all increased during 25 °C storage, age gelation only occurred in three of the samples (147 °C/6 s, 142 °C/6 s, and 142 °C/3 s treatment). Furthermore, there were significant differences in the microstructures and gel strengths among these gels. Among the gels that formed under 147 °C/6 s treatment had the densest network structure, highest gel strength, and highest water retention. Compared to the other two groups of samples that produced gels, the gel formed under 142 °C/6 s treatment had a looser structure, higher porosity, and lower water retention. The values of the porosity and water holding capacity in the gel formed under 142 °C/3 s treatment were higher than the gel formed under 142 °C/6 s treatment but lower than the gel formed under 147 °C/6 s treatment. This result was consistent with the gel strength results. The key forces maintaining the stability of the three-dimensional network structure in the gels were disulfide bonds and hydrophobic interactions. However, there were significant differences in the contributions of intermolecular interaction forces in the different gels. When the plasmin activity was high, the milk tended to form a protein sediment rather than a gel. According to the findings from this study, formation mechanisms of aging gel in direct UHT skim milk treated in different heating conditions can be proposed, as schematically illustrated in Figure 7. The results provide a basis for precise control of the process parameters of direct UHT milk and further targeted inhibition of gel formation with storage. In addition, this study was conducted on a pilot scale and has the potential to scale up to the industrial level. 

## Figures and Tables

**Table 1:** Changes in pH value, particle size, and zeta-potential of milk before and after direct UHT treatment.

Samples	C*	pH Value	Size (nm)	Zeta-Potential (mV)
Raw milk	--	6.75 ± 0.01 ^a^	168 ± 0 ^f^	−26.4 ± 0.2 ^de^
153 °C/0.25 s	0.03	6.72 ± 0.01 ^cde^	185 ± 0 ^b^	−25.5 ± 0.2 ^ab^
147 °C/0.25 s	0.02	6.71 ± 0.01 ^de^	187 ± 1 ^a^	−25.8 ± 0.2 ^bcd^
142 °C/0.25 s	0.01	6.71 ± 0.00 ^cd^	182 ± 1 ^c^	−26.7 ± 0.1 ^e^
157 °C/0.116 s	0.02	6.72 ± 0.00 ^cd^	183 ± 1 ^bc^	−25.5 ± 0.3 ^bc^
153 °C/0.116 s	0.01	6.71 ± 0.01 ^ef^	189 ± 1 ^a^	−25.9 ± 0.3 ^bcd^
147 °C/6 s	0.47	6.71 ± 0.01 ^def^	175 ± 0 ^d^	−26.3 ± 0.6 ^cde^
142 °C/6 s	0.33	6.70 ± 0.01 ^f^	172 ± 1 ^e^	−26.0 ± 0.3 ^bcde^
147 °C/3 s	0.24	6.73 ± 0.01 ^bc^	176 ± 2 ^d^	−26.6 ± 0.4 ^de^
142 °C/3 s	0.16	6.71 ± 0.01 ^ef^	182 ± 2 ^c^	−25.3 ± 0.3 ^ab^
153 °C/3 s	0.37	6.73 ± 0.01 ^b^	172 ± 1 ^e^	−24.7 ± 0.6 ^a^

^a–f^ Results are mean ± SD (*n* = 3). For each row, different letters indicate significantly different values (*p* < 0.05).

**Table 2:** Water holding capacity and porosity of age-gels formed in direct UHT milk samples after storage at 25 °C.

	Water Holding Capacity (%)	Porosity (%)
147 °C/6 s	95.1 ± 0.4 ^a^	14.3 ± 0.6 ^a^
142 °C/6 s	59.9 ± 1.8 ^c^	42.4 ± 0.8 ^c^
142 °C/3 s	79.5 ± 3.4 ^b^	21.5 ± 1.8 ^b^

^a–c^ Results are mean ± SD (*n* = 3). For each row, different letters indicate significantly different values (*p* < 0.05).

## Data Availability

The data presented in this study are available on request from the corresponding author. The data are not publicly available due to [privacy].

## Supplementary Materials

The following supporting information can be downloaded at https://www.mdpi.com/article/10.3390/foods13081236/s1. Table S1: Heating conditions and storage temperatures of all samples. Figure S1. Sedimentation in all samples during the later period of storage.
