# Supplementary material for: Age Gelation in Direct Steam Infusion Ultra-High-Temperature Milk: Different Heat Treatments Produce Different Gels
Source: Foods. 2024 Apr 18;13(8):1236. doi: 10.3390/foods13081236 (PMC11049407; doi:10.3390/foods13081236)
Supplement: Supplementary file 1 [file foods-13-01236-s001.zip › foods-2936900-supplementary.pdf]

Table S1. Heating conditions and storage temperatures of all samples

Sample[1]	Preheating condition	direct UHT temperature (°C)	Heating time (s)	C*	Direct system	Storage temperature (°C)
Raw	——	——	——		——	
1		153	0.25	0.03		
2		147	0.25	0.02		
3		142	0.25	0.01		
4		157	0.116	0.02		
5	75°C/25 s	153	0.116	0.01	Steam infusion	4, 25, 37
6		147	6	0.47		
7		142	6	0.33		
8		147	3	0.24		
9		142	3	0.16		
10		153	3	0.37		

[1] The sample number in the table was the batch of samples in the factory.

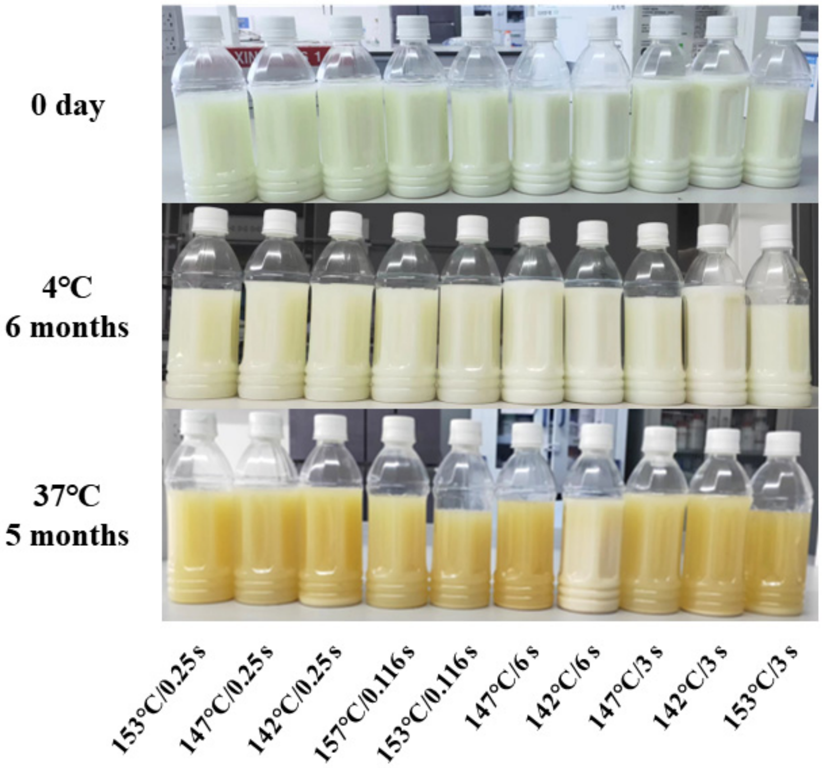

Figure S1. Sedimentation in all samples during the later period of storage
